# Psychometric properties of the modified Suicide Stroop Task (M-SST) in patients with suicide risk and healthy controls

**DOI:** 10.3389/fpsyg.2024.1332316

**Published:** 2024-03-14

**Authors:** Helena Gold, Maria Stein, Heide Glaesmer, Lena Spangenberg, Maria Strauss, Georg Schomerus, Katarina Stengler, Juliane Brüdern

**Affiliations:** ^1^Department of Medical Psychology and Medical Sociology, University of Leipzig, Leipzig, Germany; ^2^Department of Clinical Psychology and Psychotherapy, University of Bern, Bern, Switzerland; ^3^Translational Research Center, University Hospital of Psychiatry and Psychotherapy, University of Bern, Bern, Switzerland; ^4^Department of Psychiatry and Psychotherapy, University of Leipzig, Leipzig, Germany; ^5^Department of Psychiatry, Psychotherapy and Psychosomatics, Helios Park Hospital Leipzig, Leipzig, Germany

**Keywords:** suicide attentional bias, behavioral assessment, Suicide Stroop Task, implicit risk marker, psychometric properties, suicide

## Abstract

The Cognitive Model of Suicide proposes a suicide attentional bias in individuals with suicidal thoughts and behavior (STBs). The Suicide Stroop Task (SST) was developed as a behavioral measure to assess this attentional bias. However, prior studies demonstrated poor psychometric properties of the SST.

**Methods:**

We developed a modified Suicide Stroop Task (M-SST) and tested its psychometric properties in a sample of healthy controls (*n* = 30) and inpatients with STBs (*n* = 24). Participants (50% female, aged 18 to 61 years) completed the M-SST with neutral, positive, negative, suicide-related positive and suicide-related negative words. Interference scores were calculated by subtracting the mean reaction time (mean RT) of the neutral words from the mean RT of the suicide-related positive words (mean RT_Suicide-Positive_–mean RT_Neutral_) and suicide-related negative words (mean RT_Suicide-Negative_–mean RT_Neutral_), resulting in two suicide-specific interference scores. Similarly, interference scores were calculated for the positive and negative words by subtracting the mean RT of neutral words from the mean RT of positive and negative words.

**Results:**

When analyzed separately, patients with STBs showed greater interferences for suicide-related positive words (*p* = 0.039), and for suicide-related negative words (*p* = 0.016), however, we found no group differences in interference scores for positive and negative words, suggesting a suicide attentional bias in patients with STBs. Controlling for the repeated measure design, a repeated measure ANOVA failed to detect a significant group × interference interaction effect (*p* = 0.176), which limits the generalizability of the findings. However, the interference score of suicide-related negative words showed an adequate classification accuracy (AUC = 0.72, 95% CI [0.58–0.86], *p* = 0.006) for differentiating between healthy controls and patients with STBs. Moreover, the interference scores showed acceptable internal reliability for the total sample and only suicide-related interference scores were correlated with clinical characteristics, thus demonstrating convergent validity.

**Conclusion:**

The results provide preliminary evidence for a suicide attentional bias in individuals with STBs compared to healthy controls. The M-SST represents a promising tool for assessing a suicide attentional bias by revealing adequate psychometric properties. Future studies with larger samples are needed to confirm these preliminary findings.

## 1 Introduction

Every year, more than 700,000 people worldwide die by suicide, a loss that has further profound impacts on their families, society and the economy (World Health Organization, 2021). Despite increased research efforts that aim to improve the detection and prediction of suicidal thoughts and behaviors (STBs), the accuracy of suicide risk prediction has not improved significantly over the last 50 years (Large et al., 2016; Franklin et al., 2017). At present, self-report measures, such as the Beck Scale for Suicide Ideation (Beck and Steer, 1993), are a commonly used method for assessing the risk of STBs (Kleiman et al., 2023). However, Busch et al. (2003) found that most patients who died by suicide while in hospital or directly after discharge did not report suicidal ideation during their last contact with a clinician. This may have several reasons: for example, patients in clinical settings may conceal suicidal thoughts due to perceived negative consequences or they may lack insight into their own risk status. Another reason may be the absence of suicidal ideation at the moment of the assessment, as studies using ecological momentary assessment (EMA) indicate that suicidal thoughts highly fluctuate over the course of a few hours (Kleiman et al., 2017; Hallensleben et al., 2018; Brüdern et al., 2022). Accordingly, several studies have shown that the use of risk scales failed to predict suicide attempts (Steeg et al., 2018), and the Self-harm Guideline of the UK National Institute of Clinical Excellence advices against the use of risk scales for predicting suicide [National Institute for Health and Care Excellence (NICE), 2022]. Therefore, in recent years there has been an increasing interest in enhancing the detection and prediction of STBs by using behavioral measures. The latter offer the opportunity of capturing implicit cognitive processes that run automatically and might be less affected by conflicting intentions than self-report questionnaires (Anestis and Green, 2015; Richards et al., 2019).

A promising approach for investigating such implicit processes is to assess selective attention toward suicide-specific stimuli. This suicide-specific attentional bias is theoretically linked with the Cognitive Model of Suicide proposed by Wenzel and Beck (2008). The model assumes that individuals with an activated suicide schema have difficulties disengaging from relevant suicide-related information (e.g., suicide-related words) because the confrontation with such stimuli activates a suicide-specific network including cognitive processes (suicidal thoughts) and associated emotions, and prevents the person from regulating their attention away from these stimuli. This is also in line with assumptions of the Dual-System Model of Suicidality (DSMS; Brüdern et al., 2022). The DSMS posits that maladaptive implicit processes such as the suicide-specific attentional bias run automatically and unconsciously, are activated in the context of situational factors (e.g., negative events, negative affect, high stress level), and prevent an adaptive emotion regulation and coping with suicidal thoughts and urges.

The Suicide Stroop Task (SST) represents a behavioral assessment tool that was developed for measuring an activated suicide-specific network, wherein an increased response latency on suicide-related words indicates a suicide attentional bias. Cha et al. (2010) developed the first computerized SST that was administered to recent suicide attempters and non-attempter psychiatric controls. The SST consisted of trials with three neutral words, three negative words, three positive words, and three suicide-related words. The trials were randomly presented on a screen in red or blue ink. Participants were asked to indicate the color by pressing a red or blue key on the computer keyboard as quickly as possible. Reaction times for each trial were recorded and a suicide-specific interference score was calculated by subtracting the mean reaction time (Mean RT) of the neutral words from the mean RT of the suicide related words (Mean RT_Suicide_–Mean RT_Neutral_). Similarly, interference scores were calculated for the positive and negative words resulting in three interference scores. They found that only suicide-related interference was significantly greater among suicide attempters than in non-attempters indicating a suicide-related attentional bias in suicide attempters. Moreover, a greater suicide-specific interference score predicted suicide attempts 6 months later, beyond other clinical risk factors.

Over the last decade, the computerized SST of Cha et al. (2010) has been tested in several studies (Chung and Jeglic, 2016, 2017; Cha et al., 2017, 2018; Stewart et al., 2017; Niu et al., 2021) which revealed mixed results. In a recent meta-analysis, Wilson et al. (2019) investigated the psychometric properties of the SST. By comparing suicide attempters vs. suicide ideators vs. non-suicidal controls (healthy and patient controls), the SST demonstrated poor psychometric properties and failed to show a significant difference in suicide interference between the two- and three-group comparisons. Due to the lack of group differences in previous studies, some authors started generally questioning whether a suicide-attentional bias did indeed exist in individuals with STBs (Moscardini and Tucker, 2023).

### 1.1 Study aims

Given the mixed evidence regarding a suicide attentional bias, the first aim of the present study was to investigate a suicide attentional bias in patients with STBs compared to healthy controls by using a modified version of the SST (M-SST). In this regard, we developed an additional positive suicide-related word category in order to investigate whether positive aspects of the suicide-specific schema (e.g., relief) are also able to activate a suicide-related attentional bias in patients with STBs. Consequently, the M-SST consists of a negative suicide-related word category and a positive suicide-related word category. According to the Cognitive Model of Suicide (Wenzel and Beck, 2008), we hypothesized that patients with STBs show a significantly greater interference for suicide-related positive and suicide-related negative words compared to healthy controls. However, patients with STBs and controls should not differ in their interferences for positive and negative words, indicating an attentional bias only for suicide-related words in individuals with STBs.

Due to the insufficient psychometrics of the current SST, the second aim of the study was to improve the psychometric properties by considering several recommendations of previous SST studies (e.g., Wilson et al., 2019). Therefore, we applied a block-wise design, increased the number of category-specific stimuli, and used a microphone instead of keys for measuring reaction times with the aim of reducing potential cognitive interference due to possible key searching behavior. For the verification of the word material used in the M-SST, we assessed how strongly participants felt aroused by each word category and how positively versus negatively they evaluated each word. These data provide detailed information on the suitability of word stimuli.

## 2 Materials and methods

### 2.1 Participants and procedure

The study sample consisted of *n =* 24 psychiatric inpatients (*M* = 31.71 years, *SD* = 13.01) with recent suicidal ideation and *n =* 30 healthy controls (*M* = 30.30 years, *SD* = 9.30), who were well matched on age and gender (see Table 1). All participants were White. The included psychiatric inpatients were admitted to psychiatric emergency wards in a German hospital due to an acute suicidal crisis (acute suicidal ideation or a current suicide attempt). Suicidal patients indicated a mean time of 7.25 days of having experienced suicidal thoughts prior to the assessment (*SD* = 10.88) measured by a short version of the Suicidal Thoughts and Behaviors Interview (SITBI-G; Fischer et al., 2014). Patients differed significantly in all clinical characteristics from controls, which is due to the exclusion criterion of having no previous or current mental disorder in healthy controls. Diagnoses were collected from inpatients’ medical records from the psychiatric ward. Inpatients were excluded if they presented with an acute psychotic disorder. The most common diagnoses according to the International Classification of Diseases (World Health Organization, 2021) were affective disorders (F3: *n* = 14; 58.33%), neurotic, stress-related and somatoform disorders (F4: *n* = 5; 20.38%), and personality disorders (F6: *n* = 5; 20.83%). Of the suicidal patients group, 41.67% (*n* = 10) reported recent suicide ideation with no lifetime suicide attempt, 25% (*n* = 6) reported one suicide attempt, and 33.33% (*n* = 8) multiple suicide attempts. Further sample characteristics are summarized in Table 1. The control group was recruited via flyers from the surrounding communities. Participants of the control group were excluded, if they had experienced lifetime or recent suicidal ideation or a suicide attempt, a mental disorder or had undergone psychotherapy, which was checked with the Short Version of the German Structured Clinical Interview for Mental Disorders (Mini-DIPS; Margraf and Cwik, 2017). Further exclusion criteria for both groups included inability to speak or write German fluently, presence of cognitive impairment, color blindness, and dyslexia.

**Table 1:** Sociodemographic and clinical characteristics of the sample.

Variable	Control group *n* = 30 *M (SD)*	Suicidal patients *n* = 24 *M (SD)*	Test
Age (in years)	30.30 (9.30)	31.71 (13.01)	t(52) = 0.45, *p* = 0.645
Gender (%)			χ^2^ (2) = 3.55^a^, *p* = 0.175
Female Male Non-binary	53.5 46.7 0	45.8 41.7 12.5	
BDI	3.20 (2.81)	36.79 (11.68)	t(52) = 15.25, *p* < 0.001
BHS	2.77 (2.13)	14.96 (5.41)	t(52) = 11.32, *p* < 0.001
BSS Intelligence, MWT-B	0.00 (0.00) 29.80 (3.99)	19.17 (8.50) 30.13 (2.98)	t(52) = 12.38, *p* < 0.001 t(52) = 0.33, *p* = 0.741

BDI, Beck Depression Inventory; BHS, Beck Hopelessness Scale; BSS, Beck Scale for Suicide Ideation. ^a^Fisher’s exact test due to cell frequencies under 5.

All participants were informed about the purpose of the study, the voluntary nature of their participation and data storage, and gave written informed consent before participating. A research assistant provided information about the study to the suicidal patients after receiving consent from their attending psychiatrist. If patients gave informed consent, an appointment for the M-SST session was scheduled and participants received self-report questionnaires, which they had to complete before the M-SST session. The session took place in the research assistant’s office on the psychiatric ward. First, patients completed the computerized M-SST followed by an evaluation of the presented word stimuli with the Suicide Stroop Survey (see measures). Finally, a vocabulary test (Lehrl, 2005) was administered to screen for cognitive impairment, followed by a short version of the SITBI-G (Fischer et al., 2014) for assessing patients’ STBs history.

Participants of the control group were screened over the telephone regarding the exclusion criteria. If they were eligible for study participation, we sent self-report questionnaires to them by post and made an appointment for the M-SST session. Control participants took the M-SST in the lab of the Department of Medical Psychology and Medical Sociology at the University of Leipzig. The procedure was identical to that for patients except that the Mini-DIPS (Margraf and Cwik, 2017) was administered to the controls instead of the SITBI-G (Fischer et al., 2014). Every participant received 25 € for compensation. All procedures were approved by the ethic committee of the Medical Faculty of the University of Leipzig [473/20-ek].

### 2.2 Measures

Beck Depression Inventory (BDI-II). The German version of the revised Beck Depression Inventory (Beck et al., 1996; Kühner et al., 2007) was used to assess the severity of depression over the last 2 weeks. The BDI-II contains 21-items describing depressive symptoms that are to be rated on a 4-point scale (0 to 3). Total scores range from 0 to 63 with higher scores indicating greater depression. The internal consistency in our sample was high with Cronbach’s α = 0.98.

Beck Hopelessness Scale (BHS). Hopelessness was assessed with the German version of the Beck Hopelessness Scale (Beck et al., 1988; Kliem and Brähler, 2016) which comprises 20 true-false items (rated 0 or 1) that assess hopeless and pessimistic cognitions. Good reliability and validity have been shown for the BHS (McMillan et al., 2007). Total scores range from 0 to 20 with higher scores indicating stronger hopelessness. The internal consistency in our sample was high with Cronbach’s α = 0.96.

Beck Scale for Suicide Ideation (BSS). Suicidal ideation during the past week was assessed using the German version of the Beck Scale for Suicidal ideation (Beck and Steer, 1993; Kliem et al., 2017). The BSS consists of 21 statement groups and is used to assess the severity of suicidal symptoms on a 3-point scale (0 to 2). Two filter questions (the statement groups four and five) assess the presence of active or passive suicidal thoughts. If participants endorse one of them (i.e., chose a sentence rated 1 or 2), they are to complete the subsequent 14 statement groups which allow for an assessment of the severity of existing suicidal ideation. If participants choose the response option rated “0” for both item 4 and item 5 they skip items 6 to 19 and precede to the last two statement groups. These last two items address frequency and intensity of former suicide attempts and are again to be answered by all participants. They are not part of the total BSS score. The scale has shown good internal consistency and construct validity (Kliem et al., 2017). Total scores range from 0 to 38 with higher scores indicating greater suicidality. The internal consistency (calculated by using the total scores) in our sample was high with Cronbach’s α = 0.83.

Self-Injurious Thoughts and Behaviors Interview (SITBI-G). The German version of the Self-Injurious Thoughts and Behaviors Interview (Nock et al., 2007; Fischer et al., 2014) is a structured interview and assesses the frequency and intensity of the patients’ suicidal thoughts, plans and behavior. It was applied to the patients group in order to collect information regarding STBs history. The SITBI-G has good interrater and retest reliability, as well as good convergent validity (Fischer et al., 2014).

Multiple Choice Vocabulary Test, version B (MWT-B). The MWT-B (Lehrl, 2005), a vocabulary-intelligence test, was administered to assess the participant’s intelligence and was used to screen for cognitive impairment. It consists of 37 items with a maximum score of 37. Scores were transformed into IQ-values, whereby higher scores reflect higher intelligence. Every item consists of five words of which four words are non-words. Participants had to select the correct word in every item.

Short Version of the German Structured Clinical Interview for Mental Disorders (Mini-DIPS). The Mini-DIPS (Margraf and Cwik, 2017) is a short diagnostic interview for mental disorders. It was used in the control group to verify that the participants had no history of a mental disorder or STBs.

Modified Suicide Stroop Task (M-SST). For measuring a suicide-specific attentional bias, we developed the Modified Suicide Stroop Task (M-SST) by considering several recommendations from prior SST research (e.g., block-wise design, increasing the number of word stimuli). For the M-SST, we used the E-Prime 3.0 software and the response and stimulus device Chronos (Psychology Software Tools, Inc, 2023). The M-SST consists of five different word categories: neutral words, positive words, negative words, positive suicide-related words, and negative suicide-related words. To select eligible words for the suicide-related positive and suicide-related negative category, we screened suicide online forums in order to identify words that were positively or negatively associated with suicide. Subsequently, we presented a preselection of words to experts in the field of suicide research and clinicians, who evaluated each word regarding its emotional relevance to patients with SBTs. Based on this evaluation, we conducted a final selection of word stimuli. Each word category consists of 10 nouns, which were controlled regarding the number of letters and number of syllables (see Supplementary Table S1). The 10 words of each category were presented in four different font colors (e.g., the word “suicide” was presented in red, yellow, blue, and green) resulting in 40 trials (10 words in four colors) for each word category. The 40 trials of each word category were presented block-wise resulting in five experimental blocks á 40 trials: a block with neutral words, a block with positive words, a block with negative words, a block with positive suicide-related words, and a block with negative suicide-related words. All stimuli were presented on a gray screen of a DELL Latitude Laptop with a screen diameter of 15.6 inches. Participants were instructed to say the font color of the displayed word as quickly and accurately as possible into a microphone, which was connected with the Chronos device. The Chronos device measured the reaction time in milliseconds and provided an audio file with the recorded answer for each trial. Prior to starting the M-SST, a microphone test containing 20 trials (stimuli consisted of words with clothes, e.g., jacket) was conducted in order to test the microphone settings. After the microphone test, the M-SST started with 20 practice trials (words describing music instruments) followed by the five experimental blocks. For the experimental blocks of the M-SST, four different block orders were developed, which were randomly distributed across participants in order to avoid position and sequence effects.

Each trial started with the presentation of a “+” in the center of the screen for 500 milliseconds (ms) followed by the stimulus. The stimulus was displayed on the screen until the microphone registered the participant’s answer. Each trial was limited to a maximum response time of 4,000 ms. If no response was registered within this time frame, the reaction time for this trial was automatically set to zero and the trial was excluded. The time between trials was set to 1,000 ms. Between each experimental block, participants had a rest of 30 s before the next block started automatically. During the administration of the M-SST, the experimenter was blind to the block order and manually registered incorrect responses (naming the wrong font color or reading the word) by using a blind checkbox. Trials with incorrect responses were excluded from the analysis. Outlier response times were defined as response latencies <200 ms (Mogg and Bradley, 2002; Munafò et al., 2003) and were excluded before calculating the mean reaction times and interference scores.

Suicide Stroop Survey (SSS). Following the M-SST, the word material used was evaluated by the participants using the SSS that was developed by our research team. Participants had to evaluate each word of each category regarding its emotional arousal (“How much did the word affect you emotionally?”) as well as its positive (“How positively do you rate the following words?”) and negative valence (“How negatively do you rate the following words?”). The emotional arousal items were rated on a 10-point Likert scale from 1 (*not at all*) to 10 (*very strong*). The same scale was used for the negative and positive valence items from 1 (neutral/not positive or *neutral*/*not negative*) to 10 (*very positive* or *very negative*). The internal consistency in our sample was high with Cronbach’s α = 0.92.

### 2.3 Statistical analyses

Descriptive characteristics of the sample were analyzed using t-tests for continuous data, χ^2^-tests for dichotomous data, or Fisher’s exact test, if cell frequencies were smaller than 5.

As described in the measures section, trials with incorrect responses were excluded from the analysis. Outlier response times were defined as response latencies <200 ms (Mogg and Bradley, 2002; Munafò et al., 2003) and were excluded before calculating the mean reaction times and interference scores We decided against the data cleansing procedure used by Cha et al. (2010) and other SST studies (see Wilson et al., 2019; Moscardini and Tucker, 2023) which removed trials with RTs ± two standard deviations from that participants mean RT, and trials with mean RTs ± two standard deviations from the group mean RT. The procedure has been criticized as a critical limitation of prior SST research because it increases the risk of eliminating meaningful data and decreases the probability of detecting significant effects (Wilson et al., 2019; Moscardini and Tucker, 2023).

#### 2.3.1 Group differences in interference scores

For determining group differences, we used interference scores as dependent variables consistent with prior SST research (Cha et al., 2010; Wilson et al., 2019; Niu et al., 2021). For calculating interference scores, each participant’s raw RTs of the valid trials were averaged, which yielded mean RTs for each word-category specific block (information on means and standard deviations of the mean RTs are included in the Supplementary Table S2). Interference scores were computed by subtracting each participant’s mean RT of the block with neutral words (which is referred as the non-emotional reference category) from their mean RT of the block with positive words (= Interference_Positive_), the block with negative words (= Interference_Negative_), the block with suicide-related positive words (= Interference_Suicide-Pos_), and the block with suicide-related negative words (= Interference_Suicide-Neg_).

Group differences in interference scores we calculated in two stages. First, to test our hypothesis that healthy controls would differ in suicide-related positive and suicide-related negative, but not positive or negative, interference, we analyzed each interference score separately, consistent with previous SST research (Cha et al., 2010; Stewart et al., 2017; Niu et al., 2021; Moscardini and Tucker, 2023). Specifically, we tested group differences (controls versus patients) on positive, negative, suicide-related positive, and suicide-related negative interference scores using independent samples *t*-tests. To control for the repeated measure design, as a second step, we computed a repeated measure analysis of variance (ANOVA) with *group* as the between-subject factor (i.e., controls, patients) and *interference* as the within-subject factor for detecting a group × interference interaction. In this model, a significant group × interference interaction indicates that group effects significantly vary across interference type.

#### 2.3.2 Psychometric properties of the M-SST

For determining the psychometric properties of the M-SST, we estimated AUC values (area under the curve) using receiver operating characteristics (ROC) analyses for the interference scores as a classification metric of the M-SST.

Furthermore, we computed the internal reliability of the M-SST by calculating the split-half reliability (odd- vs. even-numbered trials) with Spearman-Brown correction as in Wilson et al. (2019) across the total sample (*n* = 54) as well as within the control group (*n* = 30) and patients group (*n* = 24) for interference scores. Additionally, we calculated the correlations between the interference scores and clinical self-report measures across the total sample using Pearson’s correlation coefficients, whereas correlations between interference scores and the number of lifetime suicide attempts were computed for the patients group only.

For evaluating the utility of the word material assessed with the SSS, we followed the steps applied for calculating group differences in interference scores. First, we conducted independent samples *t*-tests for each word-category (neutral, positive, negative, suicide-related positive, suicide-related negative) for the subscales arousal, positive valence, and negative valence. Subsequently, we conducted repeated measure ANOVAs for arousal, positive valence, and negative valence with *group* as the between-subject factor and a five level within-subject factor for *word category* (neutral words, positive words, negative words, suicide-related positive words, and suicide-related negative words) for detecting interaction effects.

*p*-values below 0.05 were deemed to be statistically significant. All analyses were performed using SPSS (Version 29.0).

## 3 Results

*N* = 54 participants completed the M-SST, and 117 incorrect trials (1.08%) and 232 trials (2.15%) with an outlier response time were removed.

### 3.1 Group differences in interference scores

Regarding group differences in interference scores (see Table 2), suicidal patients had significantly greater interferences for suicide-related positive words, *t*(52) = 2.15, *p* = 0.039, *d* = 0.63, and for suicide-related negative words, *t*(52) = 2.58, *p* = 0.016, *d* = 0.74, compared to healthy controls. Patients and controls did not significantly differ in their positive and negative interference scores (*ps* > 0.05).

**Table 2:** Group differences in interference scores.

Score	Suicidal patients *n* = 24		Control group *n* = 30		*t*(52)	*p*	Cohen’s d	*M*		*SD*	*M*		*SD*
Interference_Positive_	13.28	79.08	2.46	50.89	0.61	0.545	0.17
Interference_Negative_	12.67	102.65	−6.68	62.08	0.81^a^	0.422	0.24
Interference_Suicide-Pos_	45.92	99.44	−2.52	53.44	2.15^a^	0.039	0.63
Interference_Suicide-Neg_	50.90	81.84	3.67	44.92	2.54^a^	0.016	0.74

M, mean; SD, standard deviation. Interference_Positive_, Interference score of positive words; Interference_Negative_, Interference score of negative words; Interference_Suicide-Pos_, Interference score of suicide-related positive words; Interference_Suicide-Neg_, Interference score of suicide-related negative words. All interference scores and standard deviations are reported in milliseconds (ms). ^a^t value reported for unequal variance.

The results of the repeated measure ANOVA for controls versus patients revealed a significant main effect for *group F*(1, 52) = 4.32, *p* = 0.043, η_p_^2^ = 0.08, but no significant *group* × *interference* interaction *F*(3, 156) = 1.67, *p* = 0.176, η_p_^2^ = 0.03.

### 3.2 Psychometric properties of the M-SST

We conducted classification metrics using area under the curve (AUC) and 95% confidence intervals for interference scores in order to classify between healthy controls and patients with STBs, which were as follows: AUCpositive words = 0.52, 95% CI [0.36, 0.68], *p* = 0.794; AUCnegative words = 0.52, 95% CI [0.35–0.68], *p* = 0.855; AUCsuicide-positive words = 0.62, 95% CI [0.47–0.78], *p* = 0.121, and AUCsuicide-negative words = 0.72, 95% CI [0.58–0.86], *p* = 0.006, indicating that the interference for suicide-related negative words showed a good classification accuracy in distinguishing patients with STBs from healthy controls. The split-half reliability (see Table 3) was acceptable for the total sample (range = 0.67–0.76), but not for the control group (range = 0.43–0.66). For patients with STBs, the reliability of the interference scores was good (range = 0.79–0.87).

**Table 3:** Split-half reliability for interference scores.

Score	All subjects *n* = 54	Control group *n* = 30	Suicidal patients *n* = 24
Interference_Positive_	0.67	0.45	0.82
Interference_Negative_	0.73	0.63	0.82
Interference_Suicide-Pos_	0.76	0.43	0.87
Interference_Suicide-Neg_	0.76	0.66	0.79

Correlations of interference scores with self-report questionnaires and intercorrelations between interference scores for the patient groups are presented in Table 4. The interference score for suicide-related positive words was significantly positively associated with suicidal ideation (BSS). The interference score for suicide-related negative words showed significant positive correlations with depression (BDI), hopelessness (BHS), and suicidal ideation (BSS).

**Table 4:** Correlations with clinical characteristics and intercorrelations between scores.

Score	BDI	BHS	BSS	lifetime suicide attempts^a^	1^a^	2^a^	3^a^
1. Interference_Positive_	0.02	0.01	0.09	−0.28	–		
2. Interference_Negative_	0.07	0.03	0.04	−0.26	0.60^**^	–	
3. Interference_Suicide-Pos_	0.14	0.24	0.30^*^	−0.26	0.30	0.42^*^	–
4. Interference_Suicide-Neg_	0.30^*^	0.31^*^	0.31^*^	−0.22	0.32	0.70^***^	0.55^**^

Correlations of the interference Scores with clinical characteristics across the total sample (*N* = 54) and correlations with lifetime suicide attempts and between interference scores for the patients group only (*n* = 24). BDI, Beck Depression Inventory; BHS, Beck Hopelessness Scale; BSS, Beck Scale for Suicide Ideation. ^a^Correlations were computed for the patients group only. ^*^p < 0.05; ^**^p < 0.01; ^***^p < 0.001.

Regarding the evaluation of the M-SST stimuli, results revealed that controls and patients were not differently aroused by neutral and positive words (*ps* > 0.05), however, patients were significantly more emotionally aroused by negative words, *t*(52) = 3.11, *p* = 0.003, *d* = 0.85, suicide-related positive words, *t*(52) = 2.92, *p* = 0.005, *d* = 0.80, and suicide-related negative words, *t*(52) = 5.17, *p* < 0.001, *d* = 1.42. This was confirmed by a significant *group* × *word category* interaction effect in the repeated measure ANOVA, *F*(4, 208) = 12.71, *p* < 0.001, η_p_^2^ = 0.20.

For the positive valence ratings, controls and patients did not significantly differ in their evaluation for neutral, positive, negative words, and suicide-related positive (*ps* > 0.05). However, patients did evaluate suicide-related negative words significantly more positively compared to controls, *t*(51) = 2.35, *p* = 0.023, *d* = 0.65. The repeated measure ANOVA revealed no significant *group* × *word category* interaction *F*(4, 208) = 1.80, *p* = 0.131, η_p_^2^ = 0.03.

For the negative valence ratings, controls and patients did not significantly differ in their evaluation for neutral, negative, suicide-related positive, and suicide-related negative words (*ps* > 0.05). However, patients did evaluate positive words significantly more negatively compared to controls, *t*(51) = 2.85, *p* = 0.006, *d* = 0.79. The repeated measure ANOVA revealed no significant *group* × *word category* interaction, *F*(4, 208) = 1.54, *p* = 0.191, η_p_^2^ = 0.03.

## 4 Discussion

The first aim of the present study was to investigate a suicide attentional bias in patients with STBs compared to healthy controls by using a modified Suicide Stroop Task (M-SST). Notably, this is the first study that aimed to explore whether positive aspects of the suicide-specific schema are able to activate a suicidal attentional bias in suicidal individuals, by including an additional suicide-related positive word category in the test. When interferences were analyzed separately, patients with STBs showed significantly greater interferences for suicide-related positive and suicide-related negative words compared to healthy controls with medium effect sizes. However, we found no significant group differences for interferences of positive and negative words, indicating preliminary evidence of a suicide attentional bias in patients with STBs. In the repeated measures ANOVA, there was a medium effect of *group*, which was not qualified by a *group* × *interference* interaction, indicating that individuals with STBs showed greater interferences for emotional stimuli regardless of word category, which limits the generalizability of our findings.

However, with regard to our second aim of examining the psychometric properties of the M-SST, the interference score of suicide-related negative words was able to adequately differentiate between patients with STBs and healthy controls, whereas positive and negative words were not better than chance in differentiating controls from suicidal patients. Therefore, our results provide preliminary evidence of a suicide attentional bias specifically for suicide-related negative words in patients with STBs, thereby supporting the Cognitive Model of Suicide (Wenzel and Beck, 2008), outlining that a suicide attentional bias serves as an implicit cognitive marker of suicidal vulnerability. Our findings further indicate that also “positive” suicide-related associations (e.g., peace, tranquility) in combination with the word “suicide” impaired attentional control in patients with STBs, although weaker compared to suicide-related negative words, which adds relevant knowledge to suicide-specific information processing in individuals with STBs.

With regard to internal reliability, the interference scores revealed acceptable to good reliability for the total sample and patients group, but not for the control group. One reason for the reduced reliability in controls might be the reduced covariance of interferences of this group (see standard deviations in Table 2), indicating a more homogeneous subgroup compared to the patients group, leading to lower correlations and thus a reduced reliability. Compared to the unacceptably low internal reliability of the interference scores of the SST reported by Wilson et al. (2019), the reliability of the M-SST has been considerably improved.

In contrast to prior SST studies (Stewart et al., 2017; Niu et al., 2021; Moscardini and Tucker, 2023), the interference score of the suicide-related negative stimuli of the M-SST demonstrated convergent validity, as it was significantly related with self-report questionnaires of depression, hopelessness, and suicidal ideation, whereas the interference scores for positive and negative words were not related to clinical variables. We also found a positive association for the suicide-related positive word category with self-reported suicidal ideation, but not with depression and hopelessness. The missing associations of this category with depression and hopelessness might be related to the “optimistic” facets of this category with regard to suicide (e.g., salvation, relief, escape). The category’s content might indicate hope of escaping an unbearable state via a suicide attempt in individuals with STBs, and therefore, self-report questionnaires of depression and hopelessness might not be appropriate convergent measures for this aspect. Furthermore, we found no significant associations between the two suicide-related categories and the number of lifetime suicide attempts in individuals with STBs. This is in line with related findings of prior SST studies (Chung and Jeglic, 2016; Moscardini and Tucker, 2023) which found no significant differences in a suicide attentional bias between participants with a history of suicidal behavior compared to those who endorsed suicide ideation but never considered taking their own life.

Regarding divergent validity of the M-SST in the clinical sample, interference scores of the suicide-related positive and suicide-related negative words were significantly associated with the interference score of negative words in patients with STBs. Although we have made a considerable effort to ensure that both suicide-related categories, but especially the suicide-related negative category, do not share an overlapping content with negative words, results suggest that both suicide-related categories have a certain common content with the negative word category, which can be viewed as a limitation of the M-SST and could be a target for further adaptations. However, results of the interference score group comparisons revealed that individuals with STBs are able to control attention for negative stimuli, whereas they showed a deficit in attentional control for suicide-related stimuli, indicating that the suicide-related categories have a specific content that impairs attentional control.

This is the first study that additionally administered an evaluation of the used word material of a Suicide Stroop Task in order to check for group differences of arousal as well as for negative and positive valence ratings. We found an interaction effect for the arousal rating showing that the arousal by different word categories varied significantly between patients and controls. In detail, patients were more aroused by negative, suicide-related positive, and suicide-related negative words compared to the control group, with the largest effect for suicide-related negative words. This pattern suggest that patients were more aroused by negative and suicide-related stimuli compared to controls. However, effect sizes indicate, that patients were most aroused by suicide-related negative stimuli, which fits well with the assumptions of the Cognitive Model of Suicide (Wenzel and Beck, 2008), assuming a cognitive vulnerability for suicide-related information. This was confirmed by our finding that patients did evaluate suicide-related negative words significantly more positively compared to controls. Altogether, the participant’s evaluation of the used word material provides valuable information for analyzing and optimizing (e.g., deleting stimuli with a low arousal in patients with STBs) behavioral assessment tools, such as the M-SST, and we recommend including such an assessment in future research.

## 5 Limitations

Results of the current study should be interpreted with some limitations in mind. First, the sample size of the present study was small and findings should be considered as preliminary. Furthermore, we tested the M-SST in a German sample and participants were rather young in age, meaning the results of the present study may not be generalizable to diverse populations and participants of older age. Second, we did not sample for nonsuicidal control groups with high psychopathological symptoms (e.g., nonsuicidal depressive individuals) and thus, we were unable to determine if a suicide attentional bias is uniquely related to STBs or can also be explained by psychiatric symptoms in general. Moreover, we did not sample for suicide-related subsamples (e.g., suicide ideators without suicide attempts (SAs) vs. suicide ideators with SAs) and consequently, we were unable to estimate the degree to which task performance differs between subsamples with different characteristics of STBs. Third, self-report measures were not completed during the M-SST session meaning that the day of the M-SST session is not necessarily included in the scores of depression, hopelessness, and suicidal ideation potentially leading to a confound of convergent validity. However, completing self-report measures prior to the M-SST may have resulted in greater endorsement of suicidal ideation confounding M-SST performance, whereas completing self-report measures after the M-SST could have confounded scores of self-report measures by an activated suicide schema. Future research should address possible confounding effects by counterbalancing the design. Fourth, we used a microphone instead of keys for measuring reaction times with the aim of reducing potential cognitive interference due to possible key searching behavior. However, this method might be less standardized with regard to the measurement of reaction times and incorrect answers, and future studies should compare the psychometric properties of the M-SST using keys. Finally, no prospective suicide attempt data was included in the present study precluding our ability to determine the predictive validity of suicide-related interference of the M-SST in relation to future suicide attempts.

## 6 Conclusion and future directions

In this initial validation study, we found preliminary evidence for a suicide attentional bias in patients with STBs compared to healthy controls regarding suicide-related positive and suicide-related negative words. More important, the M-SST demonstrated adequate psychometric properties and future studies with larger samples should aim to replicate these findings. At the same time, future research may consider certain refinements and modifications of the M-SST, for example, investigating to what extent psychometric properties change when performing the M-SST with keys instead of using a microphone in order to achieve a higher test standardization. If a more standardized M-SST also proves psychometrically sound, behavioral measures such as the M-SST can be applied for clinical use and in studies with Ecological Momentary Assessment (EMA) for examining the temporal change of implicit information processing biases that are related to suicidal vulnerability. A growing body of EMA research reveals that suicidal ideation and associated risk factors, such as negative affect, highly fluctuate over the course of a few hours and demonstrate significant temporal instability (Kleiman et al., 2017; Hallensleben et al., 2018). Gaining insights into the between- and within-person dynamic of implicit risk processes such as a suicide attentional bias would improve our understanding of a complex problem like suicidality. Along these lines, future research should also investigate if attentional control is modifiable in patients with STBs as it has already been shown in nonsuicidal cohorts with depression (Holas et al., 2020) and in high suicide risk Veterans receiving a Mindfulness-Based Cognitive Therapy (Chesin et al., 2021). All these promising future research directions require a psychometrically valid assessment tool of suicide attentional bias, with this study having taken a further step in that direction.

## Data availability statement

The raw data supporting the conclusions of this article will be made available by the authors, without undue reservation.

## Ethics statement

The studies involving humans were approved by ethic committee of the Medical Faculty of the University of Leipzig. The studies were conducted in accordance with the local legislation and institutional requirements. The participants provided their written informed consent to participate in this study. [473/20-ek].

## Author contributions

HGo: Formal analysis, Investigation, Writing – original draft. MS (second author): Supervision, Writing – review & editing. HGl: Resources, Supervision, Writing – review & editing. LS: Writing – review & editing. MS (fifth author): Resources, Writing – review & editing. GS: Resources, Writing – review & editing. KS: Writing – review & editing. JB: Conceptualization, Methodology, Supervision, Writing – original draft.
