# Supplementary material for: Psychometric properties of the modified Suicide Stroop Task (M-SST) in patients with suicide risk and healthy controls
Source: Front Psychol. 2024 Mar 14;15:1332316. doi: 10.3389/fpsyg.2024.1332316 (PMC10977103; doi:10.3389/fpsyg.2024.1332316)
Supplement: Supplementary file 1 [file Table_1.DOCX]

**Table S1**

Word Material of the M-SST

| Neutral words | Positive words | Negative words | Suicide-related positive words | Suicide-related negative words |
| --- | --- | --- | --- | --- |
| chair (Stuhl) | security (Sicherheit) | meanness (Gemeinheit) | suicide (Selbstmord) | suicide (Selbstmord) |
| fridge (Kühlschrank) | trust (Vertrauen) | jealousy (Eifersucht) | sleep (Schlaf) | despair (Verzweiflung) |
| towel (Handtuch) | luck (Glück) | dispute (Streit) | silence (Stille) | fight (Kampf) |
| shower curtain (Duschvorhang) | hope (Hoffnung) | offense (Beleidigung) | exit (Ausweg) | wish to die (Todeswunsch) |
| desk (Schreibtisch) | strengths (Stärke) | laziness (Faulheit) | rescue (Rettung) | pain (Schmerz) |
| stove (Herd) | ambition (Ehrgeiz) | difficulty (Schwierigkeit) | freedom (Freiheit) | destruction (Vernichtung) |
| door handle (Türklinke) | confidence (Zuversicht) | hostility (Feindschaft) | salvation (Erlösung) | self-hate (Selbsthass) |
| bookshelf (Bücherregal) | friendship (Freundschaft) | damage (Schaden) | relaxation (Entspannung) | end of life (Lebensende) |
| drawer (Schublade) | honesty (Ehrlichkeit) | mistrust (Misstrauen) | relief (Erleichterung) | leave (Abschied) |
| tap (Wasserhahn) | passion (Leidenschaft) | bad luck (Pech) | peace of mind (Seelenfrieden) | failure (Versagen) |

*Note.* The words in brackets are the original German words used in the M-SST. Each word category consisted of ten category-specific words, which were controlled regarding number of letters and number of syllables.
