# Supplementary material for: Psychometric properties of the modified Suicide Stroop Task (M-SST) in patients with suicide risk and healthy controls
Source: Front Psychol. 2024 Mar 14;15:1332316. doi: 10.3389/fpsyg.2024.1332316 (PMC10977103; doi:10.3389/fpsyg.2024.1332316)
Supplement: Supplementary file 2 [file Table_2.DOCX]

**Table S2**

Group Differences in Mean Reaction Times for each Category-Specific Block

|  | Suicidal patients  *n* = 24  *M SD* | | Control group  *n* = 30  *M SD* | | *t*(52) | *p* | Cohen´s d |
| --- | --- | --- | --- | --- | --- | --- | --- |
| MeanRT_Neutral_ | 808.33 | 116.19 | 699.05 | 138.16 | -3.10 | .003 | .85 |
| MeanRT_Positive_ | 821.61 | 140.84 | 701.52 | 130.04 | -3.25 | .002 | .89 |
| MeanRT_Negative_ | 821.01 | 140.91 | 692.37 | 119.80 | -3.63. | < .001 | .99 |
| MeanRT_Suicide-Pos_ | 854.25 | 178.14 | 696.53 | 154.23 | -3.49 | .001 | .96 |
| MeanRT_Suicide-Neg_ | 859.23 | 143.86 | 702.72 | 143.89 | -3.97 | < .001 | 1.10 |

*Note.* Mean RT_Neutral_ = mean reaction time for the block of neutral words, Mean RT_Positive_ = mean reaction time for the block of positive words, Mean RT_Negative_ = mean reaction time for the block of negative words, Mean RT_Suicide-Pos_ = mean reaction time for the block of suicide-related positive words, Mean RT_Suicide-Neg_ = mean reaction time for the block of suicide-related negative words, *M* = mean, *SD* = standard deviation. All means and standard deviations are reported in milliseconds (ms).
